# The spleen as a possible source of serine protease inhibitors and migrating monocytes required for liver regeneration after 70% resection in mice

**DOI:** 10.3389/fcell.2023.1241819

**Published:** 2023-09-07

**Authors:** Andrey Elchaninov, Polina Vishnyakova, Maria Kuznetsova, Elena Gantsova, Viktoria Kiseleva, Anastasiya Lokhonina, Maria Antonova, Aiaz Mamedov, Anna Soboleva, Dmitry Trofimov, Timur Fatkhudinov, Gennady Sukhikh

**Affiliations:** ^1^ Laboratory of Growth and Development, Avtsyn Research Institute of Human Morphology of FSBI “Petrovsky National Research Centre of Surgery”, Moscow, Russia; ^2^ Histology Department, Medical Institute, Peoples’ Friendship University of Russia (RUDN University), Moscow, Russia; ^3^ Laboratory of Regenerative Medicine, Institute of Translational Medicine, National Medical Research Center for Obstetrics, Gynecology and Perinatology Named After Academician V.I. Kulakov of Ministry of Healthcare of Russian Federation, Moscow, Russia; ^4^ Laboratory of Molecular Research Methods, Institute of Reproductive Genetics, National Medical Research Center for Obstetrics, Gynecology and Perinatology Named After Academician V.I. Kulakov of Ministry of Healthcare of Russian Federation, Moscow, Russia; ^5^ Histology Department, Pirogov Russian National Research Medical University, Ministry of Healthcare of the Russian Federation, Moscow, Russia

**Keywords:** spleen, liver, regeneration, macrophages, lymphocytes

## Abstract

**Introduction:** The role of the immune system in liver repair is fundamentally complex and most likely involves the spleen. The close connection between the two organs via the portal vein enables delivery of splenic cytokines and living cells to the liver. This study evaluates expression of inflammation-related genes and assesses the dynamics of monocyte-macrophage and lymphocyte populations of the spleen during the recovery from 70% hepatectomy in mice.

**Methods:** The study used the established mouse model of 70% liver volume resection. The animals were sacrificed 24 h, 72 h or 7 days post-intervention and splenic tissues were collected for analysis: Clariom™ S transcriptomic assay, immunohistochemistry for proliferation marker Ki-67 and macrophage markers, and flow cytometry for lymphocyte and macrophage markers.

**Results:** The loss and regeneration of 70% liver volume affected the cytological architecture and gene expression profiles of the spleen. The tests revealed significant reduction in cell counts for Ki-67+ cells and CD115+ macrophages on day 1, Ly6C + cells on days 1, 3 and 7, and CD3^+^CD8^+^ cytotoxic lymphocytes on day 7. The transcriptomic analysis revealed significant activation of protease inhibitor genes *Serpina3n*, *Stfa2* and *Stfa2l1* and decreased expression of cell cycle regulatory genes on day 1, mirrored by inverse dynamics observed on day 7.

**Discussion and conclusion:** Splenic homeostasis is significantly affected by massive loss in liver volume. High levels of protease inhibitors indicated by increased expression of corresponding genes on day 1 may play an anti-inflammatory role upon reaching the regenerating liver via the portal vein. Leukocyte populations of the spleen react by a slow-down in proliferation. A transient decrease in the local CD115+ and Ly6C+ cell counts may indicate migration of splenic monocytes-macrophages to the liver.

## Introduction

The immune system is heavily involved in tissue repair as a major regulatory entity complementary to nervous and endocrine elements. Clear anatomical connections between certain immune and non-immune organs ensure coherent reactions to infections, traumas, toxic damage, etc. It is primarily about the relationship between the spleen and the liver within the so-called hepatosplenic axis (Tarantino et al., 2013; Li et al., 2017). The basis of this interaction is the portal circulation linking the spleen and liver, as well as the presence of common barrier and immune functions (Tarantino et al., 2013; Li et al., 2017). Despite the direct connection of the liver and spleen, the specific mechanisms of mutual influence of these organs, including reparative processes, remain poorly understood (Yamada et al., 2016; Liu et al., 2018; Elchaninov et al., 2022).

The hepatosplenic axis was extensively studied in connection with cirrhosis in clinical and experimental models. Accordingly, the emphasis was made on spleen-derived factors affecting Ito cells of the liver implicated in the excessive fibrous matrix production (Romanelli and Stasi, 2016; Higashi et al., 2017). In hepatitis and cirrhosis, i.e., under conditions of diffuse damage to liver parenchyma, macrophages of the splenic red pulp produce increased amounts of TGFβ1 spreading to the liver via the portal vein and activating Ito cells—this effect has been confirmed in various settings, both experimental (Akahoshi et al., 2002) and clinical (Asanoma et al., 2014). By contrast, under conditions of sheer massive loss in liver volume, splenic influences appear beneficial. Increased expression of certain interleukins and hepatocyte growth factor (HGF) by the spleen after experimental liver resections (Kono et al., 1992; Yin et al., 2011) indicates a humoral support deserving a dedicated investigation.

The spleen is a plausible source of i) cytokines and growth factors that regulate inflammation and repair; and ii) migratory leukocytes. Deposition of monocytes in the spleen and their release towards the damage foci have been demonstrated in models of myocardial infarction and ischemic stroke (Swirski et al., 2009; Blomster et al., 2013; Kim et al., 2014; Rizzo et al., 2020). The immigration of monocytes-macrophages and other leukocytes in the liver after hepatotoxic injury or surgical resection is well documented (Goh et al., 2013; Zigmond et al., 2014; Elchaninov et al., 2021), but their sources and contribution of the spleen to this process are unknown.

The spleen is the largest site of T- and B-lymphocyte differentiation in adult mammals. Almost any organ in the body has its own lymphocyte population with varying degree of splenic connections (Marquez-Medina et al., 2012; Li and Hua, 2017). A decrease in CD4^+^ splenic pools has been observed in mouse model of cirrhosis (Tanabe et al., 2015) and migration to the liver as a possible cause cannot be excluded.

Reactions of splenic lymphocytes to hepatectomy have not been addressed experimentally. Moreover, in liver resection models, the hepatosplenic axis status and retrieval are often neglected. Here we address this issue using a transcriptomic approach focused on inflammation- and repair-related secreted molecules. We also assess the dynamics of monocyte-macrophage and lymphocyte populations of the spleen after 70% hepatectomy in mouse model.

## Methods

### Animals

The study used male C57bl/10 mice (*N* = 39), body weight 20–22 g, bred in “Stolbovaya” branch of the Federal State Budgetary Institution of Science “Scientific Center for Biomedical Technologies of the Federal Medical and Biological Agency.”

The animals were housed in plastic cages at 22°C ± 1 °C with light on from 6:00 a.m. to 6:00 p.m. and free access to chow and water. The conditions complied with the “International Recommendations for Conducting Biomedical Research Using Animals” of 1985, the Rules of Laboratory Practice in the Russian Federation (Order of the Ministry of Health of the Russian Federation of 91/06/2003 No. 267), and the law “On the protection of animals from abuse” chapter V, article 10, 4679-GD of 12/01/1999. The study was approved by the Ethical Review Board at the Avtsyn Research Institute of Human Morphology [Protocol No. 29 (5), 8 November 2021].

### Experimental model

Resection of 70% liver volume was performed under general isoflurane anesthesia using the Higgins and Anderson method described elsewhere (Nevzorova et al., 2015), scheduled uniformly to 10.00–11.00 a.m. The animals were sacrificed 24 h, 72 h or 7 days post-intervention using a CO2-chamber. A total of 18 hepatectomies were performed; the comparison groups included intact (*n* = 6) and sham-operated animals (*n* = 15). The process of liver regeneration took 7 days; by this time-point the differences in liver-to-body weight ratio between hepatectomized, sham-operated and intact animals levelled off (Supplementary Figure S1).

### Immunohistochemistry

Antibodies by Abcam, UK, were used in dilutions given in brackets. The scope of primary antibodies included proliferation marker Ki-67 (ab16667, 1:100) and macrophage markers CD68 (ab125212,1:100), CD163 (ab182422, 1:100) and CD206 (ab64693, 1:100).

Splenic tissue fragments were shock-frozen in liquid nitrogen and cryosectioned. The slides were incubated for 12 h with primary antibodies and for 1 h with FITC- or PE-conjugated secondary antibodies (1:200); the nuclei were counterstained with 4′,6-diamidino-2-phenylindole (DAPI, Sigma-Aldrich Co LLC). The immunostained cryosections were analyzed for positivity; the indexes were calculated as a percent of FITC-labeled cells among total cell counts, with at least 3,000 cells assessed for each target. Ki67^+^ and Ki67^-^ cells were counted in randomly selected fields of view (all cells in each field counted) until the total cell count (Ki67^+^ plus Ki67^-^) exceeded 1,000. The Ki67^+^ indexes were calculated as ratio of Ki67^+^ to the total cell count (Ki67^+^ plus Ki67^-^). The indexes were calculated for each animal individually, with red and white pulps of the spleen examined separately. The plot was built using averaged Ki67 indexes for each animal.

### Flow cytometry

The dissected splenic fragments were washed twice in Hanks’ balanced salt solution (PanEco, Russia), mechanically minced and incubated in 0.05% solution of collagenases type I and IV (PanEco) for 25 min at 37°С, shaking. The suspension was passed through 100 um nylon strainer (SPL LifeScience, Korea) and washed twice from th enzymes in Hanks’ balanced salt solution with 10% fetal calf serum (PAA Laboratories, Austria; 500 g for 10 min, 20°С). The erythrocytes were lyzed in Red Blood Cell Lysis Solution (Miltenyi Biotec, Germany).

The obtained cell preps were immunophenotyped for CD45, CD3, CD8, CD4, CD19, F4/80, CD115, Ly6C, CD163 and CD206. The cells were incubated with antibodies, washed, resuspended in 0.3 mL PBS, and transferred to flow cytometry tubes for analysis in a BD FACSAria III (Becton Dickinson, United States). Each measurement used at least 10,000 cells. The gating strategies used FSC vs. SSC dot-plots to exclude debris (Supplementary Figure S1) followed by [SSC vs. CD45 (CD45^+^ to SSC vs. CD3 (CD3^+^ to CD4 vs. CD8))] dot-plots for T cells [SSC vs. CD19 (CD19^+^ to SSC vs. CD45)] for B cells [SSC vs. CD45 (CD45^+^ to SSC vs. CD3 (CD3^+^ vs. CD1d))] dot-plots for NKT cells [SSC vs. CD45 (CD45^+^ to SSC vs. CD3 (CD3^+^ vs. CD4+to CD4 vs. Foxp3)] dot-plots for total Treg cells and [SSC vs. CD45 (CD45^+^ to SSC vs. F4/80|CD115|Ly6C)] for macrophages.

For detailed analysis of macrophage phenotypes, CD115+ cells were isolated manually by magnetic sorting using a MidiMACS™ Separator with LS Columns and Anti-CD115 MicroBeads (all by Miltenyi Biotec). CD86, CD163 and CD206 positivity indexes were measured in FSC vs. SSC gated pools of interest.

See Supplementary Table S1 for a list of antibodies for flow cytometry. The gating strategy is shown in Supplementary Figure S1.

### Transcriptomic profiling

Total RNA isolated from cells with RNeasy Plus Mini Kit (Qiagen, Germany) was analyzed by Clariom™ S Assay, mouse (Applied Biosystems™, Thermo Fisher). The data were analyzed with TAC Software 4.0.2 using Affymetrix default analysis settings and RMA algorithm. Fisher’s exact test with FDR correction was performed for estimation of statistical over-representation. The data reported in this paper have been deposited into the Gene Expression Omnibus (GEO) database, https://www.ncbi.nlm.nih.gov/geo (GEO accession: GSE232531).

### Real-time PCR

First-strand strand cDNA was synthesized with MMLV RT kit (Evrogen, Russia) using total RNA as a template. The PCRs were set in duplicates with qPCRmix-HS SYBR mastermixes containing SYBR Green I (Evrogen, Russia). The target-specific primers are listed in Supplementary Table S2. The expression was quantified by the threshold cycle (Ct) approach; relative expression levels were calculated against Gapdh as a reference target.

### Western blotting

The tissue was homogenized in protein solubilization buffer (MicroRotofor Lysis Kit, Bio-Rad) with tributylphosphine and cOmplete protease inhibitor cocktail (Roche) and centrifuged at 14,000 g for 30 min. The supernatant was mixed with sample loading buffer, heated at 65°C for 5 min and stored at −20°C. Protein separation was carried out in 10.0%–12.5% PAAG. The transfer to PVDF membranes (Bio-Rad) was performed in a Trans-Blot Turbo Transfer System (Bio-Rad) at standard settings. The membrane was incubated in EveryBlot Blocking Buffer (Bio-Rad) for 15 min at room temperature and stained with protein-specific antibodies overnight at 4°C, washed, and incubated with secondary HRP-conjugated antibodies for 1 h at room temperature. Chemiluminescence was visualized using Novex ECL kit (Invitrogen, United States) in a ChemiDoc Imaging System (Bio-Rad). Chemiluminescence intensity was analyzed using ImageLab software with GAPDH as normalization reference. The uncropped WB membranes are shown in the Supplementary Figure S2.

### Statistical analysis

The data were analyzed in SigmaStat 3.5 software (Systat Software Inc., United States) using Mann–Whitney test. The differences at *p* < 0.05 were considered statistically significant.

## Results

### Proliferation dynamics

**FIGURE 1:**
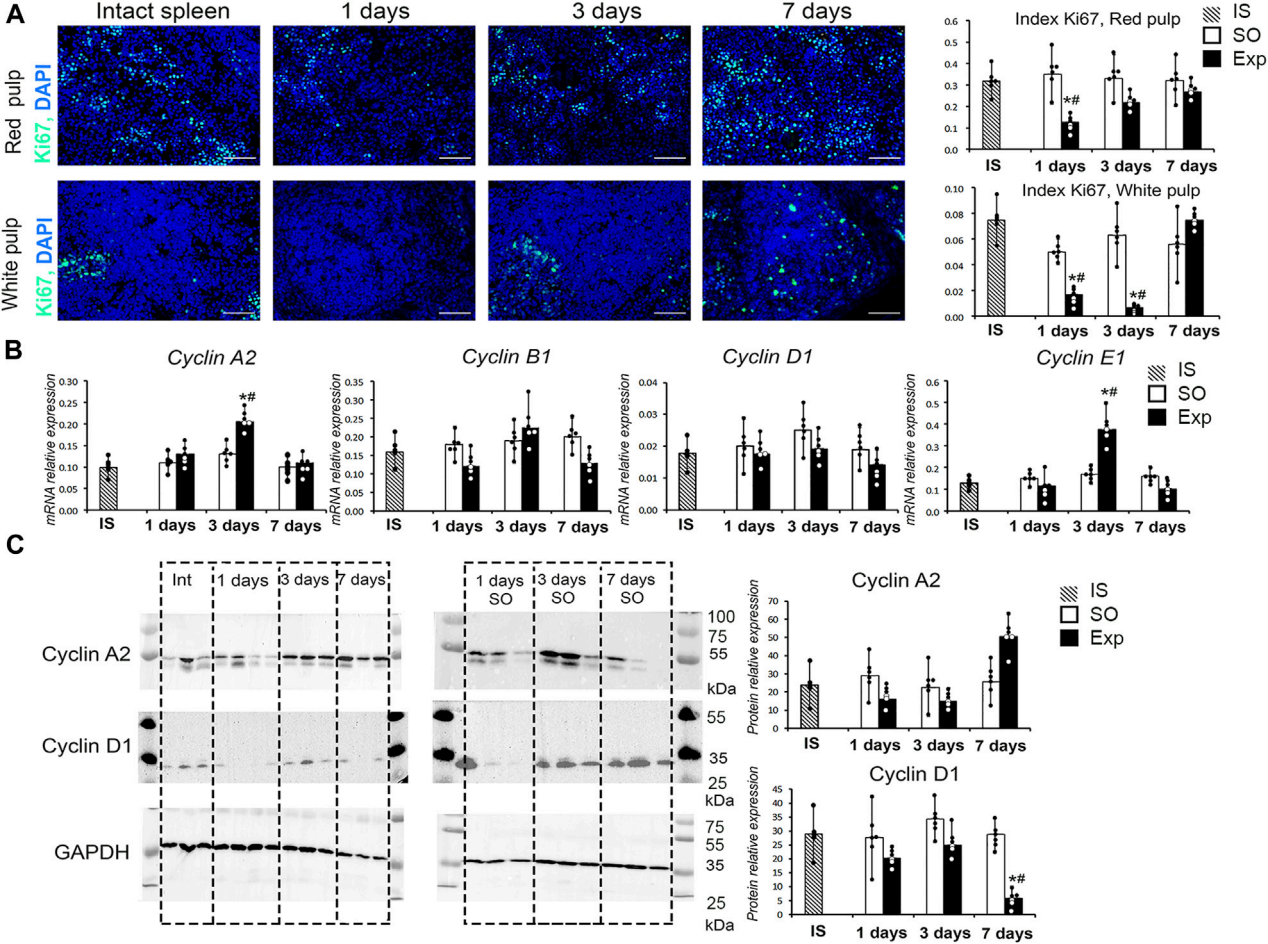
Spleen cell proliferation activity after liver resection. **(A)** Ki-67 expression in red and white spleen pulp cells after liver resection with corresponding Ki-67 indexes, FITC-conjugated secondary antibodies, cell nuclei counterstained with DAPI, objective lens 40, scale bar 50 μm **(B)** Cyclin gene expression in the spleen after liver resection. **(C)** Western blot of cyclin A2 and D1 proteins and corresponding densitometric study. IS–intact spleen, SO–sham-operated animals, EXP–animals with liver resection, the data are presented as means ± standard deviations, *p < 0.05 compared with corresponding sham-operated animals, #*p* < 0.05 compared with intact spleen.

Liver resection led to a statistically significant decrease in the proliferative activity of splenic cells. A decrease in Ki-67 index was observed in both red and white pulps of the spleen. However, in red pulp, Ki-67 index was significantly reduced on day 1 (24 h post-intervention) and returned to control values by day 3 (72 h post-intervention). In white pulp, a similar correction took longer, with Ki-67 index returning to control values by day 7 (Figure 1A). A significant increase in the expression of *Сyclin A2* and *E1* genes in the spleen was found 3 days after liver resection (Figure 1B). It should be noted that, at the protein level, a downward trend in the content of cyclin D1 in the spleen of hepatectomized animals as compared to sham-operated was revealed by Western blotting on day 7 after liver resection; in the case of cyclin A, there was a tendency for the content of this protein to increase, but the difference was insignificant (Figure 1C, Supplementary Figure S3).

### Splenic macrophage population dynamics

**FIGURE 2:**
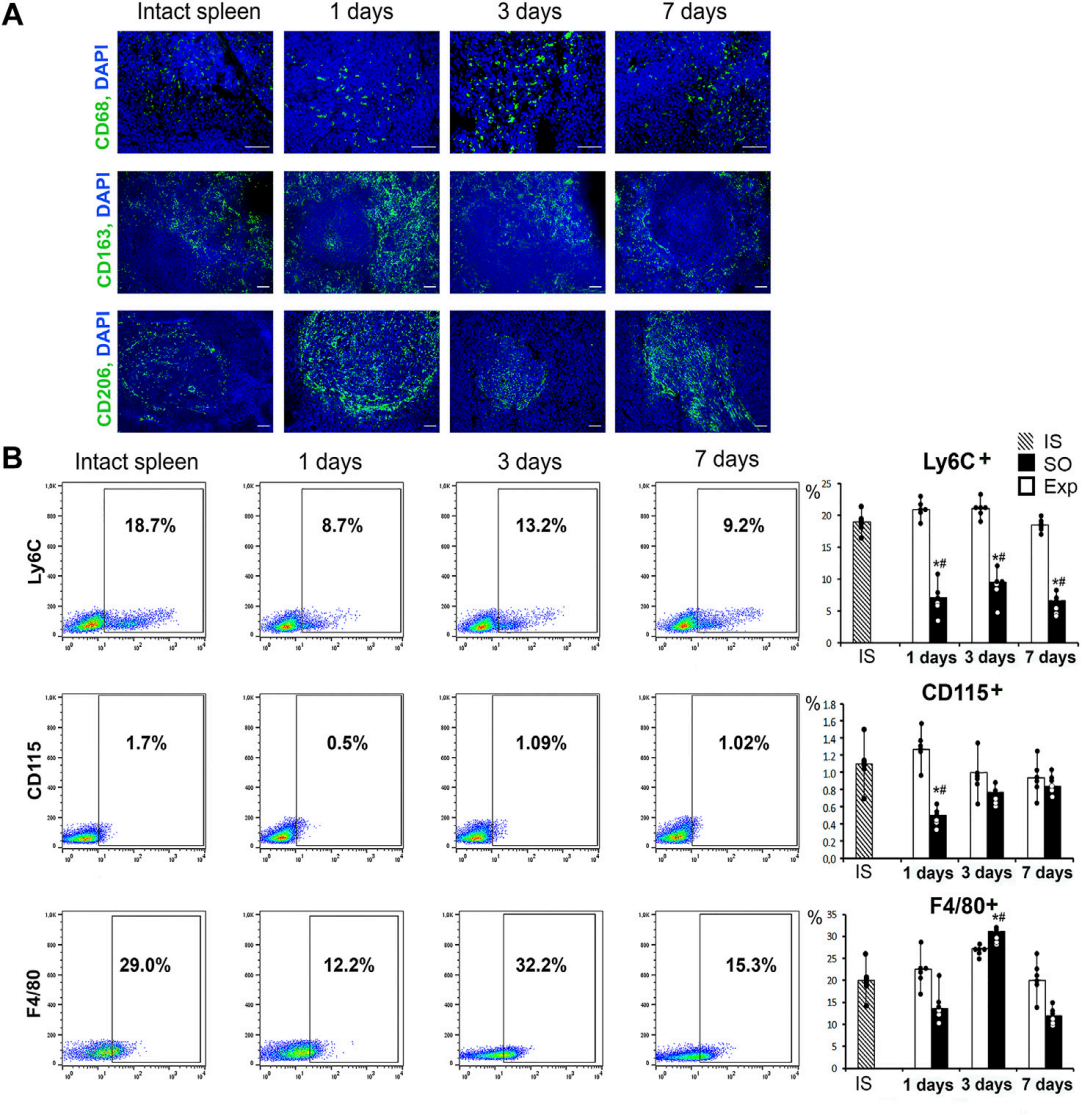
The state of the monocytic-macrophage system of the spleen after liver resection. **(A)** Immunohistochemical detection of macrophage markers CD68, CD163 and CD206, FITC-conjugated secondary antibodies, cell nuclei counterstained with DAPI. **(B)** Representative Ly6C-, CD115-, F4/80-SSC scatter plots. Reconstitution dynamics for Ly6C+, CD115+, F4/80 + cells. IS–intact spleen, SO–sham-operated animals, EXP–animals with liver resection, the data are presented as means ± standard deviations, *p < 0.05 compared with corresponding sham-operated animals, #*p* < 0.05 compared with intact spleen.

Immunohistochemical tests revealed predominant localization of CD68^+^ and CD163+ cells in red pulp, whereas CD206+ cells were predominantly associated with lymphoid follicles. This topography was characteristic of splenic macrophages in all animals independently of liver resection history. On day 1 (24 h post-intervention) CD206+ cell counts outside lymphoid follicles were increased. Also on day 1, anti-CD163 antibody produces a signal of higher intensity compared with other groups and time-points of the study (Figure 2A; Supplementary Figure S4).

**FIGURE 3:**
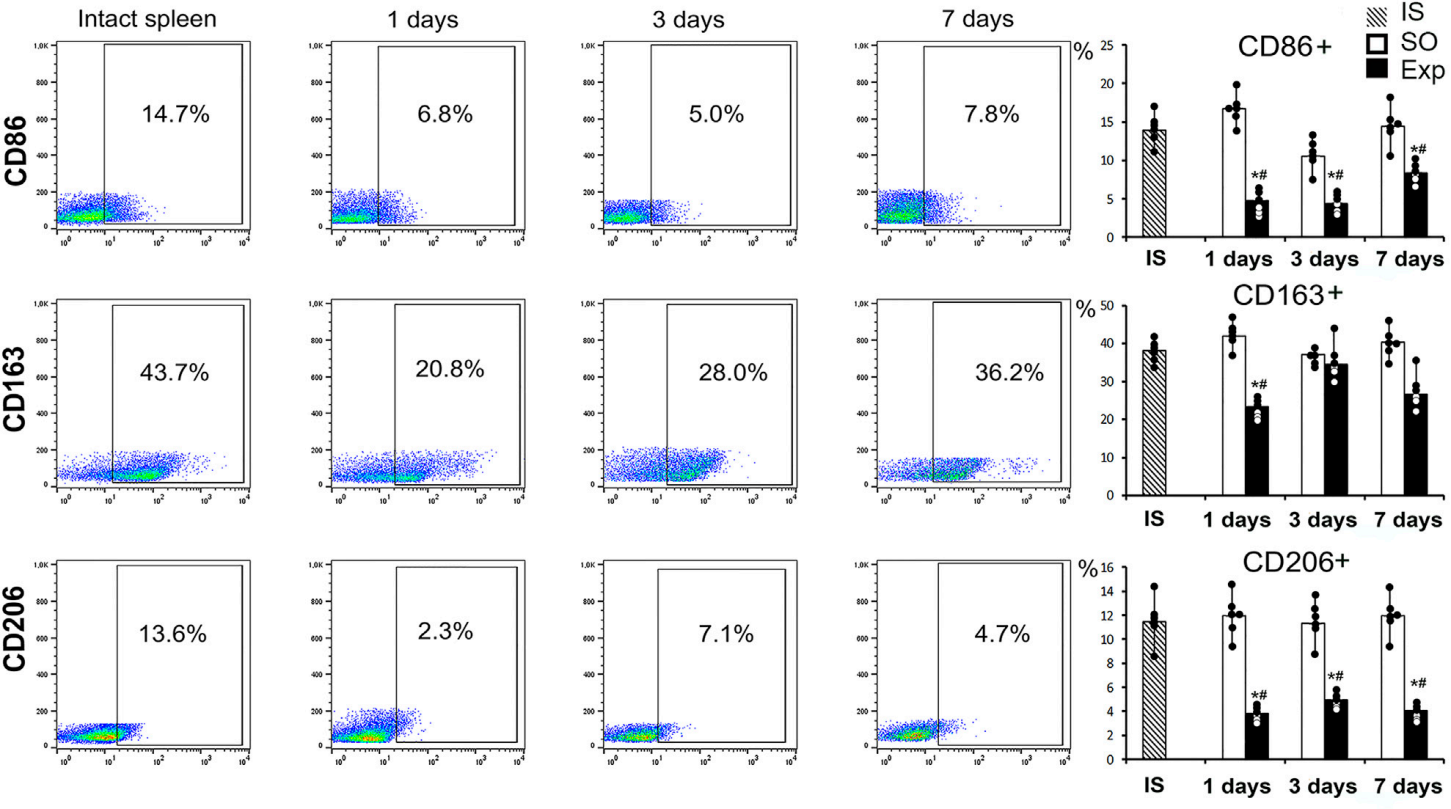
Analysis of the phenotype of macrophages obtained by magnetic sorting for the CD115 marker from the spleen after liver resection. Representative CD86^−^, CD163-, CD206-SSC scatter plots. Reconstitution dynamics for CD86^+^, CD163+, CD206 + cells. IS–intact spleen, SO–sham-operated animals, EXP–animals with liver resection, the data are presented as means ± standard deviations, *p < 0.05 compared with corresponding sham-operated animals, #*p* < 0.05 compared with intact spleen.

The flow cytometry assay revealed significant changes in splenic macrophage subpopulation ratio including decreased CD115+ counts on day 1 and decreased Ly6C+ counts on days 1, 3 and 7. On the third day after surgery we found a significant increase in the proportion of F4/80+ macrophages, which probably reflects the processes of macrophage activation in response to liver resection (Figure 2B). Splenic cell isolates sorted for CD115 macrophage surface marker showed significantly reduced content of particular subpopulations: CD163+ on day 1, CD206+ and CD86^+^ throughout the observation (days 1, 3 and 7 post-intervention) (Figure 3, Supplementary Figure S4).

### Splenic lymphocyte population dynamics

**FIGURE 4:**
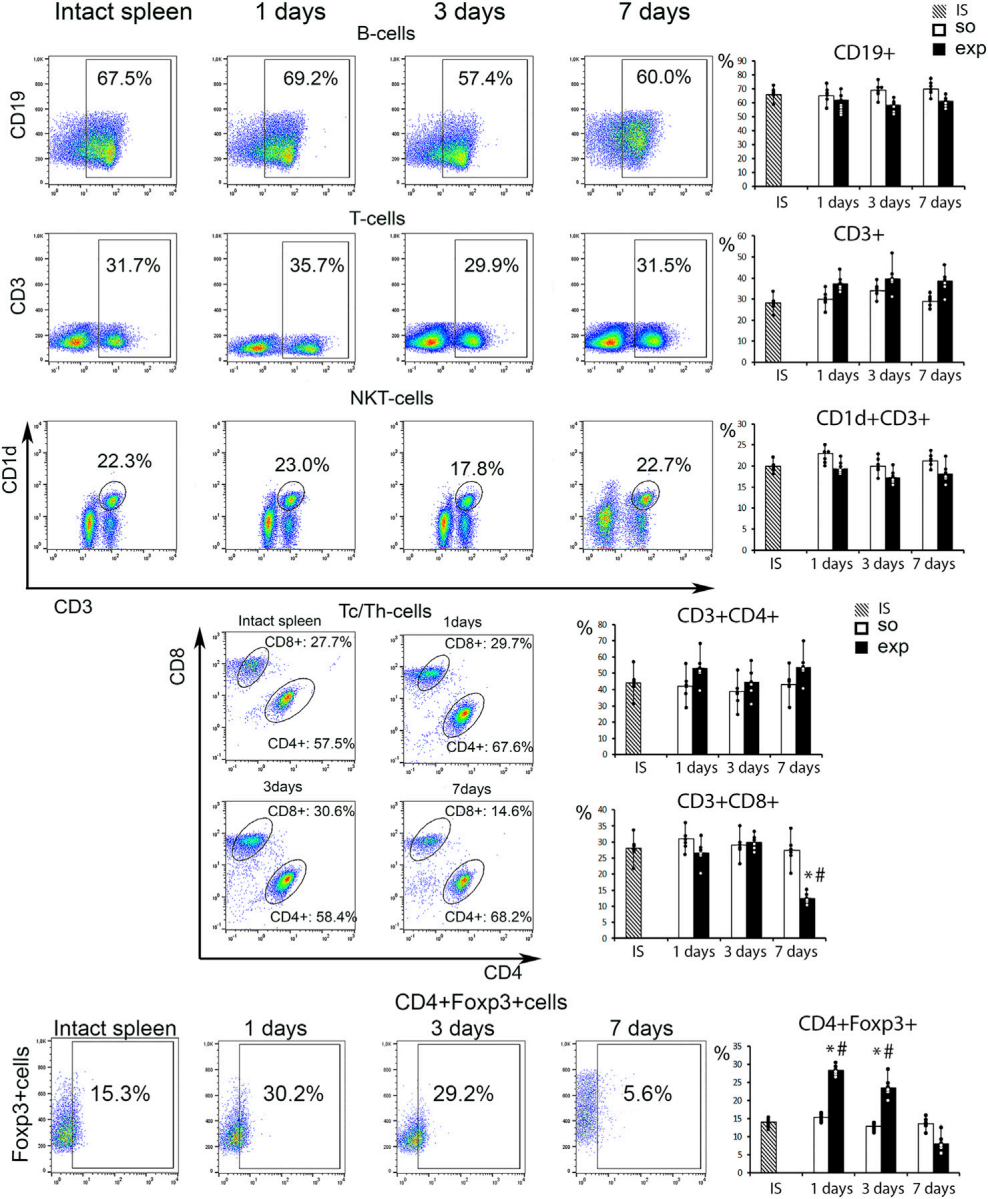
Analysis of the spleen lymphocyte population after liver resection. Representative CD19^−^, CD3^−^, CD1d vs. CD3-, CD8 vs. CD4-scatter plots. Reconstitution dynamics for CD19^+^, CD3^+^,CD1d+CD3^+^, CD3^+^CD4^+^, CD3^+^CD8+cells. IS–intact spleen, SO–sham-operated animals, EXP–animals with liver resection, the data are presented as means ± standard deviations, *p < 0.05 compared with corresponding sham-operated animals, #*p* < 0.05 compared with intact spleen.

Among various lymphocyte subpopulations assessed by flow cytometry (CD3^+^, CD19^+^, CD3^+^CD4^+^, CD3^+^CD8^+^, total T reg and NKT cells) the only one affected by the intervention were CD3^+^CD8^+^ cytotoxic lymphocytes: their counts were significantly reduced on day 7 (Figure 4, Supplementary Figure S4). Total Treg (CD4+Foxp3+) cell counts were significantly increased in hepatectomized animals 24 h and 72 h post-resection compared with the controls; the differences in Treg counts levelled off by day 7 (Figure 4; Supplementary Figure S5).

### Transcriptomic profiles

**FIGURE 5:**
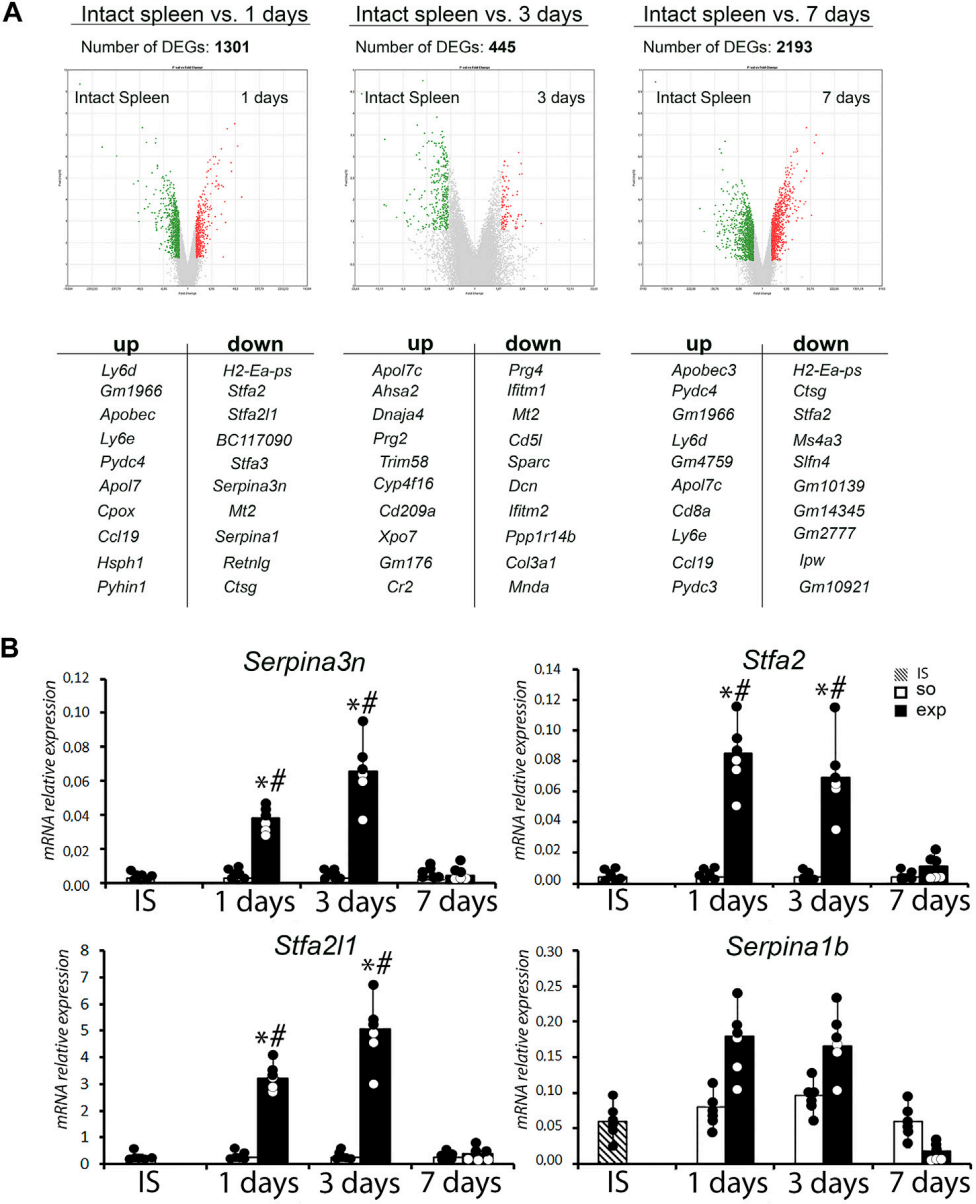
Analysis of the transcriptome of the spleen after liver resection. **(A)** Volcano plots provide a comparative overview of gene expression in intact spleen vs. 1, 3 and 7 days after liver resection with corresponding list of top 10 up-and downregulated genes. **(B)** Analysis of expression levels of a *Serpina3n*, *Stfa2*, *Stfa2l1* and *Serpina1b* genes in spleen after liver resection. IS–intact spleen, SO–sham-operated animals, EXP–animals with liver resection, the data are presented as means ± standard deviations, *p < 0.05 compared with corresponding sham-operated animals, #*p* < 0.05 compared with intact spleen.

The hepatectomy and subsequent recovery are accompanied by significant transcriptomic changes in the spleen. Numbers of differentially expressed genes (vs. controls) identified by the Clariom™ high-throughput transcriptomic assay were 1,301 on day 1; 445 on day 3; and 2,193 on day 7 (Figure 5A; Supplementary Table S3).

The enrichment analysis revealed decreased expression of cell cycle/G1-S transition-related genes (*Ccne1*, *Ccna2*, *Mcm2*, *Ccnb2*, *Rbl1*, *Cdc6*, *Ccne2*, *Plk1*) along with activation of signaling pathways related to the complement system and coagulation hemostasis (*C1qa*, *Serping1*, *Tfpi*, *C3*, *Cfh*, *C6*, *C1qb*), chemokine pathways (*Ccl9*, *Prkacb*, *Itk*, *Ccl21c*, *Cxcl12*, *Ccl6*, *Nfkbia*, *Ccl21a*, *Pik3cg*) and B cell receptor-mediated signaling (Supplementary Table S3). Apart from the acute reaction to surgical trauma combined to liver failure, the dynamics corresponds to activation of B cell lymphopoiesis, T cell migration and macrophage polarization. The highest fold changes in expression levels observed for protease inhibitor-encoding genes *Serpina3n* (56-fold ↑, compared to the intact spleen), *Stfa2* (1,019-fold ↑) and *Stfa2l1* (315-fold ↑) on day 1 plausibly reflect the immunological stress at systemic level (Figure 5A; Supplementary Table S3).

Transcriptomic profiles of the spleen on day 3 (72 h post-intervention) reflect delayed reactions to the trauma, as well as a compensatory anti-inflammatory trend. Most prominently upregulated were *Sparc*, *Cd5l*, *Mt2*, *Ifitm1* and *Prg4*. The highest number of downregulated genes belonged to mRNA processing pathways.

On day 7, virtually all upregulated genes returned to baseline expression levels, some of them going negative (*Serpina1a*, *Stfa2*, *Ctsg*). The highest enrichments were observed for B and T cell receptor signaling pathways. Increased expression of *Ccnd1* in the spleen on day 7 apparently corresponded to the completion of liver regeneration with concomitant normalization of spleen functionalities.

Transcriptional analysis data were confirmed by real-time PCR. A statistically significant increase in the expression of the *Serpina3n*, *Stfa2*, and *Stfa2l1* genes was found in the spleen 1 and 3 days after liver resection (Figure 5B).

The data reported in this paper have been deposited into the Gene Expression Omnibus (GEO) database, https://www.ncbi.nlm.nih.gov/geo (GEO accession: GSE232531).

## Discussion

The modulatory effects of the immune system on liver regeneration are a major focus in biomedical research. The spleen, which is the largest site for antigen-dependent maturation of immune cells and coordination of immune responses in the body, is close to the liver both physically and functionally, and connected to it via the portal vein. Negative effects of the spleen on functional status of the liver were demonstrated in experimental models of hepatitis and liver cirrhosis, where splenectomy in the model animals alleviated the degree of fibrotic changes to the liver (Tarantino et al., 2013; Li et al., 2017). The effect was explained as follows. Hepatitis, the leading cause of liver fibrosis, is accompanied by pervasive cell death of hepatocytes. The products of decay enter systemic circulation and eventually reach the spleen where they stimulate pro-inflammatory ad pro-fibrotic cytokine and growth factor secretion (Akahoshi et al., 2002; Asanoma et al., 2014; Tang et al., 2016). Straightforward delivery of these factors to the liver via the portal vein closes the loop; splenectomy cancels the feedback altogether (Elchaninov et al., 2022).

Splenic influences in liver regeneration after 70% resection are less evident. Although removal of the spleen was reported to negatively affect the rates of liver repair after 70% resection (Babaeva and Zotikov, 1987), mechanisms of this effect have not been elucidated. Several assumptions made in this regard can be reduced to two main possibilities: 1) the spleen is a site for the synthesis of biologically active substances that enter the regenerating liver with the bloodstream and modulate repair processes and 2) the spleen can act as a source of immune cells (monocytes, lymphocytes, etc.) migrating to the liver and contributing to its regeneration on the spot.

Studies assuming model 1) were designed based on current knowledge of secretomic influences of immune cells on tissue repair, ultimately focusing on a selection of few biologically active secreted molecules as plausible candidates. One study showed induction of hepatocyte growth factor gene expression in the spleen (Kono et al., 1992; Yin et al., 2011). Another study showed that IL17a or IL6 secreted by the spleen activate hepatocyte proliferation in the liver (Furuya et al., 2013; Behnke et al., 2018). We used a more general approach to avoid assumptions and studied the spleen transcriptome in order to comprehensively cover a variety of factors that can be produced in the spleen and regulate liver regeneration. We observed hepatectomy-induced increases in expression of serine protease inhibitor-encoding genes *Serpina3n*, *Stfa2* and *Stfa2l1*which may favorably influence the repair processes in the liver.

The role of protease inhibitors in tissue repair is a recent focus in experimental studies. In paracetamol-induced hepatotoxic injury, increased levels of Serpina3n alleviated the degree of necrotic and inflammatory changes in the liver (Tran et al., 2023) and a similar trend was observed for experimental ischemic stroke (Zhang et al., 2022). The elevated splenic levels of Serpina3n may spread via the portal vein to exert a healing, anti-inflammatory effect in the regenerating liver.

The other two differentially expressed protease inhibitors, *Stfa2* and *Stfa2l1*, can further mitigate the inflammatory response by inhibiting cysteine endo- and exopeptidases, notably cathepsins L and S involved in antigen presentation (Mihelič et al., 2006). The inhibition of cathepsin S in experimental Sjögren syndrome reduced both the autoantibody production and the lymphocytic infiltration of salivary and lacrimal glands (Saegusa et al., 2002; Roper et al., 2003).

In this section, we would like to point out some limitations of our research. When studying the spleen transcriptome during liver resection, only the spleen of intact animals was used as a control. In our opinion, this is justified by identical data obtained for intact and sham-operated animals by other analytical tools used in this study, including RT PCR, Western blot analysis and flow cytometry. As the increased expression levels of serine protease inhibitor genes *Serpina3n*, *Stfa2* and *Stfa2l1* in the spleen of hepatectomized animals is sustained for up to 7 days after the intervention, association of these changes with sheer opening-suturing of the abdominal cavity is unlikely.

Another possible mechanism of splenic influence on repair processes in the liver is based on immune cell recruitment from the spleen to the site of injury, as described for other models (Swirski et al., 2009; Blomster et al., 2013; Kim et al., 2014; Rizzo et al., 2020).

Apart from the transcriptomic influences, hepatectomy significantly altered the cytological architecture and leukocyte subpopulation balance of the spleen. The Ki-67 positivity-measured proliferation activity of splenic parenchyma was reduced on days 1 and 3 and returned to normal by day 7 post-intervention. Expression of cyclin genes followed these dynamics, albeit not at the protein level; a similar discrepancy for cyclin mRNA and protein expression levels has been reported previously (Albrecht et al., 1995). The hepatectomy-induced suppression of splenic hemopoiesis may unleash some compensatory mechanisms, as indicated by increased expression of megakaryocyte stimulating factor Prg4 known to stimulate myelo-and lymphopoiesis (Novince et al., 2011).

The efflux of monocytes from the spleen after liver resection is indirectly indicated by decrease in splenic monocyte counts. The hepatectomy-induced changes in the monocyte-macrophage subpopulation balance of the spleen include a reduction in CD115+ and Ly6C + relative cell counts. These data are consistent with previously reported dynamics attributed to recruitment of splenic monocytes to damaged organs (Swirski et al., 2009; Blomster et al., 2013; Kim et al., 2014; Rizzo et al., 2020). Accordingly, the spleen is a likely source of monocytes that colonize the remnant liver during its compensatory growth and functional recovery. In this regard, it is interesting to note the induced splenic expression of Sparc (osteonectin) involved in regulation of cell adhesion, extracellular matrix remodeling and differentiation/homing of immune cells at particular locations in the body (Riley and Bradshaw, 2020). The plausible migration of splenic monocytes to regenerating liver should be regarded as a beneficial influence, as the post-hepatectomy inhibition of the monocyte-macrophage influx to the liver impedes the regeneration (Constandinou et al., 2008; You et al., 2013).

Apart from the signs of monocyte-macrophage efflux from the spleen, we observed changes in their functional status indicated by the reduced CD86^−^, CD163-and CD206-positivity among cells expressing the M-CSF receptor CD115. The trend apparently reflects the reaction of macrophages to pro-inflammatory stimuli. A competing anti-inflammatory stimulation of splenic milieus may be indicated by increased expression of Cd5l known to encode a protein which stimulates the anti-inflammatory macrophage polarization (Sanjurjo et al., 2018).

The observed effects of liver resection on splenic lymphocyte populations were limited to a significant decrease in the proportion of cytotoxic T cells on day 7. Cytotoxic T cells are important for liver regeneration after hepatectomy: they produce lymphotoxins which stimulate IL-6 synthesis and hepatocyte proliferation (Tumanov et al., 2009). The observed decrease in the cytotoxic T cell counts inside the spleen may indicate their migration to the liver or reflect the activation of Treg cells shown to increase in number inside the spleen after liver resection. Treg cells can exert anti-inflammatory effects by several mechanisms (Li et al., 2018). They produce anti-inflammatory interleukins (IL4, IL10, IL13, IL35) and stimulate production of the same molecules by neutrophils and macrophages (Hawrylowicz and O’Garra, 2005; Taams et al., 2005; Collison et al., 2007). The anti-inflammatory cytokines inhibit the activity of CD8^+^ and CD4^+^ lymphocytes thereby supporting repair processes (Weirather et al., 2014). The anti-inflammatory effect of Treg cells apparently underlies their favorable effect on liver regeneration after resection (Wang et al., 2023).

Overall, the accumulated evidence confirms both the positive and the negative roles of the spleen in liver repair. A dominance of either negative or positive influences under particular circumstances can be explained by the nature of liver damage—whether it involves a toxicity component. In situations of pervasive hepatocyte death, exemplified by hepatitis and liver fibrosis, the spleen becomes an extra source of damage-aggravating cytokines; accordingly, its removal stimulates repair processes in the liver. In situations of massive loss and zero extraneous toxicity within the remnant, the rates of hepatocyte cell death are minimal (Elchaninov et al., 2016); accordingly, the spleen stays non-intoxicated by decay products and imposes no extra cytokine burden on the regenerating liver. Moreover, splenectomy under these conditions will negatively affect the compensatory growth of the healthy remnant by cancelling the influx of monocytes and biologically active molecules including the extracellular protease inhibitors and HGF. The negative impact of splenectomy on healthy liver tissue, both intact and regenerating after 70% resection of its volume, was demonstrated experimentally (Babaeva and Zotikov, 1987; Babaeva, 1989; Makarova et al., 2012).

The liver exchange with the bloodstream is enormous, and it is difficult to identify specific substances addition or subtraction of which by means of hepatectomy will influence gene expression profiles of splenic cells. A likely candidate is bacterial endotoxin (lipopolysachcaride, LPS) produced in the gut. The liver is the major site of LPS utilization; accordingly, endotoxin levels in the blood of hepatectomized animals are increased (Michalopoulos and DeFrances, 1997) and may promote pro-inflammatory activation of macrophages and other TLR4-expressing cells in the spleen, eventually counteracted by anti-inflammatory cascades associated with increased production of serine protease inhibitors.

Another possible mechanism of hepatic influence on other organs involves bile acids. The circulation of bile acids between the liver and the small intestine is affected by hepatectomy, as the remnant liver cannot cope with bile acid recycling and their blood levels may increase. Macrophages express bile acid receptors including the farnesoid X receptor (FXR) (Renga et al., 2009) activation of which promotes the anti-inflammatory M2 polarization of macrophages while blocking the TH1/TH17 lymphocyte inflammatory polarization (Shi et al., 2022; Jaroonwitchawan et al., 2023).

## Conclusion

Liver resection causes significant changes in the spleen, affecting its transcriptomic profiles and leukocyte homeostasis. The early stages of liver repair are accompanied by induction of certain anti-inflammatory markers, notably the protease inhibitor genes *Stfa2l1*, *Stfa2* and *Serpina3n* and partial repression of cyclin genes in the spleen. Leukocyte populations of the spleen react to hepatectomy by decreased proliferation rates and shifted subpopulation balance. Significant reduction in relative counts of CD115+ and F4/80+ cells in the spleen may indicate an increase in the monocyte-macrophage efflux.

## Data Availability

The datasets presented in this study can be found in online repositories. The names of the repository/repositories and accession number(s) can be found below: https://www.ncbi.nlm.nih.gov/geo/, GSE232531.
