# Supplementary material for: The spleen as a possible source of serine protease inhibitors and migrating monocytes required for liver regeneration after 70% resection in mice
Source: Front Cell Dev Biol. 2023 Sep 7;11:1241819. doi: 10.3389/fcell.2023.1241819 (PMC10512715; doi:10.3389/fcell.2023.1241819)
Supplement: Supplementary file 1 [file DataSheet1.docx]

Supplementary Material

The spleen as a possible source of serine protease inhibitors and migrating monocytes required for liver regeneration after 70% resection in mice

Andrey Elchaninov^1,3^*, Polina Vishnyakova^2,3^, Maria Kuznetsova^5^, Elena Gantsova^1,3^, Viktoria Kiseleva^2^, Anastasiya Lokhonina^2,3^, Maria Antonova^4^, Aiaz Mamedov^4^, Anna Soboleva^1^, Dmitry Trofimov^5^, Timur Fatkhudinov^1,3^, Gennady Sukhikh^2^

1 Laboratory of Growth and Development, Avtsyn Research Institute of Human Morphology of FSBI "Petrovsky National Research Centre of Surgery", Moscow, Russia

2 Laboratory of Regenerative Medicine, Institute of Translational Medicine, National Medical Research Center for Obstetrics, Gynecology and Perinatology Named after Academician V.I. Kulakov of Ministry of Healthcare of Russian Federation, Moscow, Russia

3 Histology Department, Medical Institute, Peoples' Friendship University of Russia (RUDN University), Moscow, Russia

4 Histology Department, Pirogov Russian National Research Medical University, Ministry of Healthcare of the Russian Federation, Moscow, Russia

5 Laboratory of molecular research methods, Institute of Reproductive Genetics, National Medical Research Center for Obstetrics, Gynecology and Perinatology Named after Academician V.I. Kulakov of Ministry of Healthcare of Russian Federation, Moscow, Russia

*** Correspondence:**Andrey Elchaninov
elchandrey@yandex.ru

**Supplementary Table 1.** List of antibodies for flow cytometry

| **Description of the antibody** | **Dilution** | **Manufacturer** | **Catalogue number** |
| --- | --- | --- | --- |
| Anti-Mouse CD45 Antibody PE | 1:100 | Miltenyi Biotec | 130102596 |
| Anti-Mouse Ly6C Antibody FITC | 1:100 | Miltenyi Biotec | 130111777 |
| Anti-Mouse F4/80 PerCP/Cyanine5.5 Conjugated | 1:100 | BioLegend | DL20130F |
| Anti-Mouse CD115 PE Conjugated | 1:100 | BioLegend | DL21294F |
| Anti-Mouse CD115 APC Conjugated | 1:100 | BioLegend | DL21295F |
| Anti- Mouse CD206 PE Conjugated | 1:100 | BioLegend | DL21672F |
| Anti-Mouse CD86 PE/Cyanine7 Conjugated | 1:100 | BioLegend | DL20106F |
| Anti-Mouse CD163 PE Conjugated | 1:100 | eBioscience | 2006963 |
| Anti-Mouse CD19 Antibody PerCP/Cyanine5.5 Conjugated | 1:100 | BioLegend | DL20011F |
| Anti-Mouse CD3 Monoclonal Antibody APC Conjugated | 1:100 | BioLegend | DL20268F |
| Anti-Mouse CD8a Antibody PE Conjugated | 1:100 | BioLegend | DL22851F |
| Anti-Mouse CD4 Antibody EV450 Conjugated | 1:100 | BioLegend | DL22845F |
| Anti-Mouse CD1d Monoclonal Antibody APC Conjugated | 1:100 | BioLegend | DL20484F |
| Anti-Mouse Foxp3 Antibody PE Conjugated | 1:100 | BioLegend | DL22662F |

**Supplementary Table 2.** Primers for real-time PCR

| Target designation | 5′-end primer | 3′-end primer |
| --- | --- | --- |
| *CcnA2* | TGTCCTGGATTGGGTCACTGG | TCAGCCTCCGGGCAGTAGA |
| *CcnB1* | GCTAAGATCAGCACGCTGGC | TCGACAACTTCCGTTAGCCTAAACT |
| *CcnD1* | TGTCGGCGCAGTAGCAGA | AAGATACGGAGGGCGCACAG |
| *CcnE1* | TGGATGGTTCCGTTCGCCAT | GTCAGGACCACACTCGGAGG |
| *Serpina3n* | CAAGCCAACAACCCTGAACATCA | GCATCCATTCCCAACGTGCC |
| *Serpina1b* | TGGGGCTGACCTCTCTGGAA | GGCATAGACATAGGAACGGCTTC |
| *Stfa2* | AGTTCAAGTCGTCCAAGGACTAAA | CGCATCTCTTTACAATGGGGGTTA |
| *Stfa2l1* | TTGTCAGAGGCCAGACCTGC | TGGAGGTAACAACCACGTCCT |
| *Gapdh* | AGGCCGGTGCTGAGTATGTC | TGCCTGCTTCACCACCTTCT |

**Supplementary Table 3.** Top 10 enriched pathways in the spleen after liver resection

**1 days**

| **Pathway** | **Total DEGs** | **Upregulated genes** | **Downregulated genes** |
| --- | --- | --- | --- |
| Protein-protein interactions in podocytes | 58 | 12 | 46 |
| Focal adhesion: PI3K-Akt-mTOR signaling pathway | 27 | 6 | 21 |
| mRNA processing | 24 | 10 | 14 |
| Chemokine signaling pathway | 24 | 9 | 15 |
| Protein-protein interactions in the podocyte | 23 | 6 | 17 |
| Mechanisms associated with pluripotency | 22 | 4 | 18 |
| Oxidative stress and redox pathway | 17 | 2 | 15 |
| Insulin signaling | 16 | 5 | 11 |
| Adar1 editing defficiency immune response | 16 | 14 | 2 |
| Mapk signaling pathway | 16 | 4 | 12 |

**3 days**

| **Pathway** | **Total DEGs** | **Upregulated genes** | **Downregulated genes** |
| --- | --- | --- | --- |
| mRNA processing | 23 | 0 | 23 |
| Electron transport chain | 18 | 0 | 18 |
| Protein-protein interactions in podocytes | 17 | 4 | 13 |
| Oxidative phosphorylation | 11 | 0 | 11 |
| Cytoplasmic ribosomal proteins | 9 | 0 | 9 |
| Protein-protein interactions in the podocyte | 8 | 1 | 7 |
| Mechanisms associated with pluripotency | 7 | 1 | 6 |
| Proteasome degradation | 6 | 0 | 6 |
| Oxidative stress and redox pathway | 6 | 0 | 6 |
| Chemokine signaling pathway | 6 | 3 | 3 |

**7 days**

| **Pathway** | **Total DEGs** | **Upregulated genes** | **Downregulated genes** |
| --- | --- | --- | --- |
| Protein-protein interactions in podocytes | 95 | 58 | 37 |
| B cell receptor signaling pathway | 47 | 40 | 7 |
| T cell receptor signaling pathway | 46 | 41 | 5 |
| mRNA processing | 40 | 9 | 31 |
| Mechanisms associated with pluripotency | 40 | 21 | 19 |
| Chemokine signaling pathway | 34 | 24 | 10 |
| Protein-protein interactions in the podocyte | 30 | 20 | 10 |
| Focal adhesion: PI3K-Akt-mTOR signaling pathway | 29 | 15 | 14 |
| Oxidative stress and redox pathway | 28 | 4 | 24 |
| Electron transport chain | 27 | 2 | 25 |


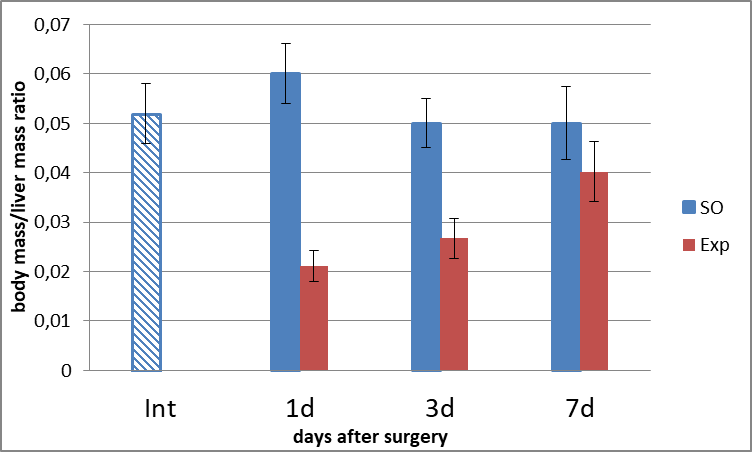


*#

*#

**Supplementary Figure 1. Dynamics of restoration of liver mass.** * - *p* < 0.05 compared with corresponding sham-operated animals, # - *p* < 0.05 compared with intact animals.

**
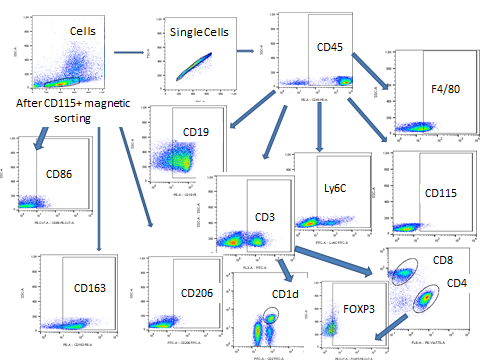
**

**Supplementary Figure 2.** The gating strategies. The gating strategies used FSC vs SSC dot-plots to exclude debris (Fig.S1) followed by (SSC vs CD45 (CD45+ to SSC vs CD3 (CD3+ to CD4 vs CD8))) dot-plots for T cells; (SSC vs CD19 (CD19+ to SSC vs CD45)) for B-cells; (SSC vs CD45 (CD45+ to SSC vs CD3 (CD3+ vs CD1d))) dot-plots for NKT cells; (SSC vs CD45 (CD45+ to SSC vs CD3 (CD3+ vs CD4))vs. CD4+to FOXP3) for total Treg cells and (SSC vs CD45 (CD45+ to SSC vs F4/80|CD115|Ly6C)) for macrophages. After CD115+magnetic sorting CD86, CD163 and CD206 positivity indexes were measured in FSC vs SSC gated pools of interest.

**Supplementary Figure 3. Uncropped WB membranes**

Cyclin D 1


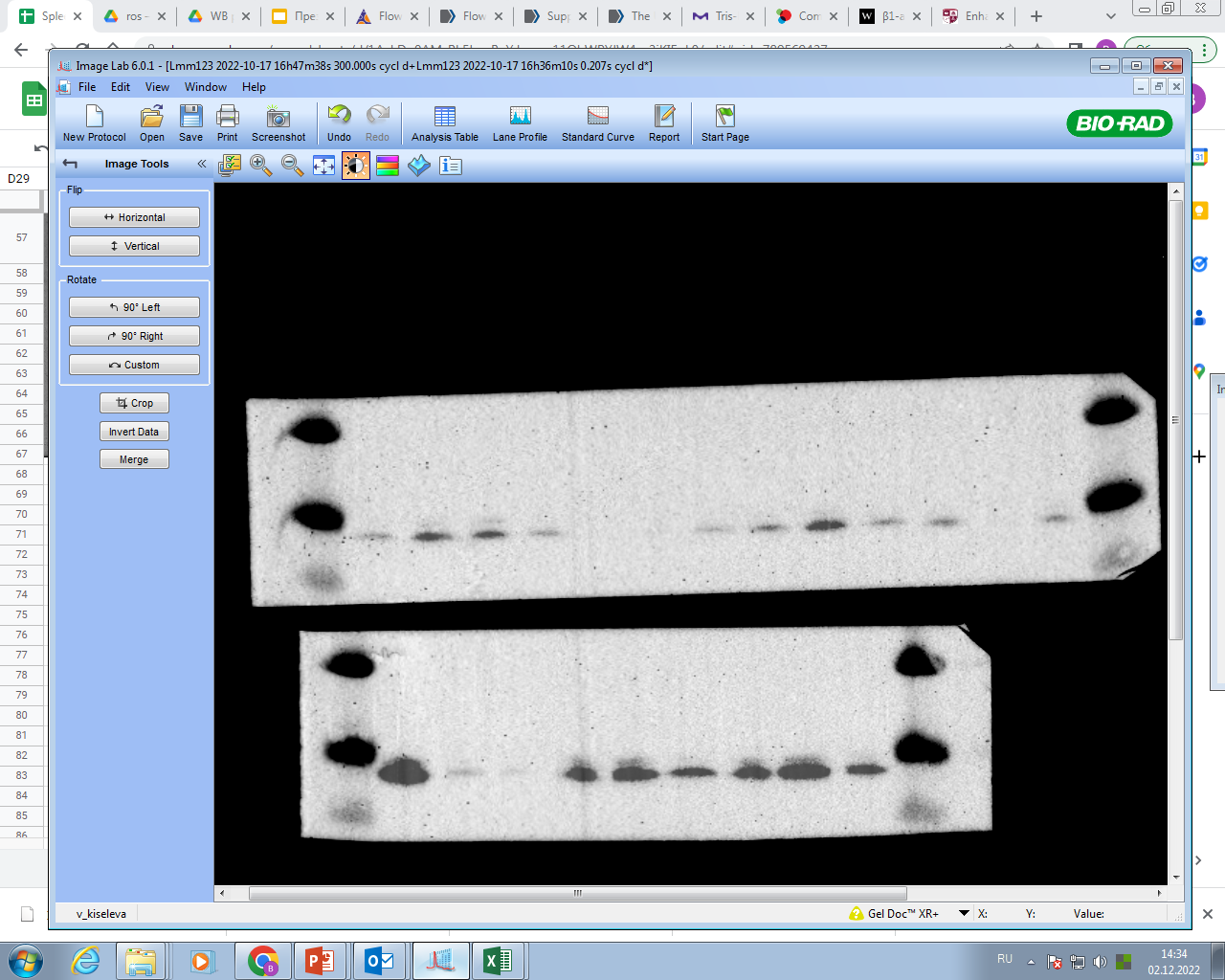


55

35

25

7 days

3 days

1 days

Intact spleen


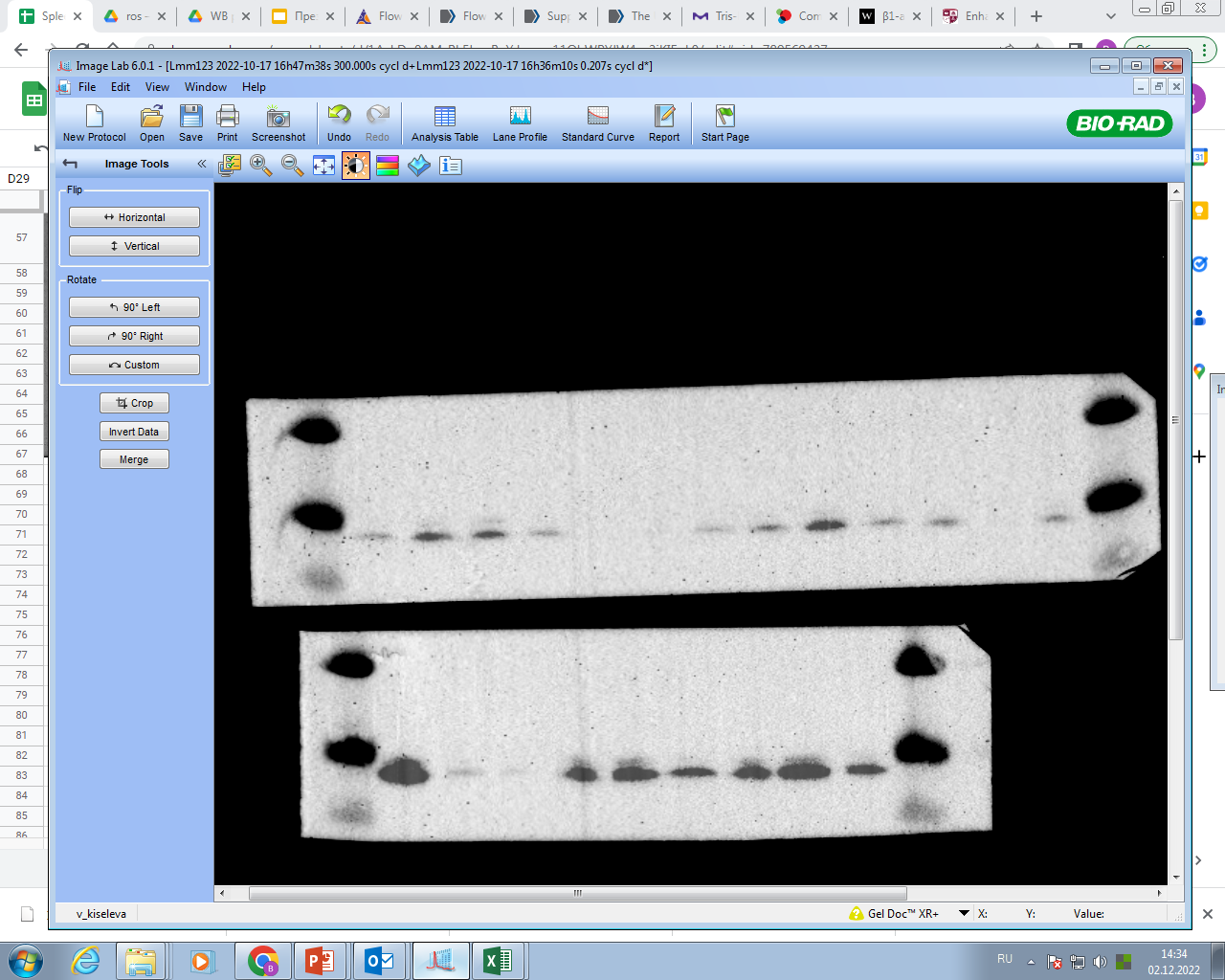


35

25

55

sham-operated

7 days

sham-operated

3 days

sham-operated

1 days

Cyclin A2


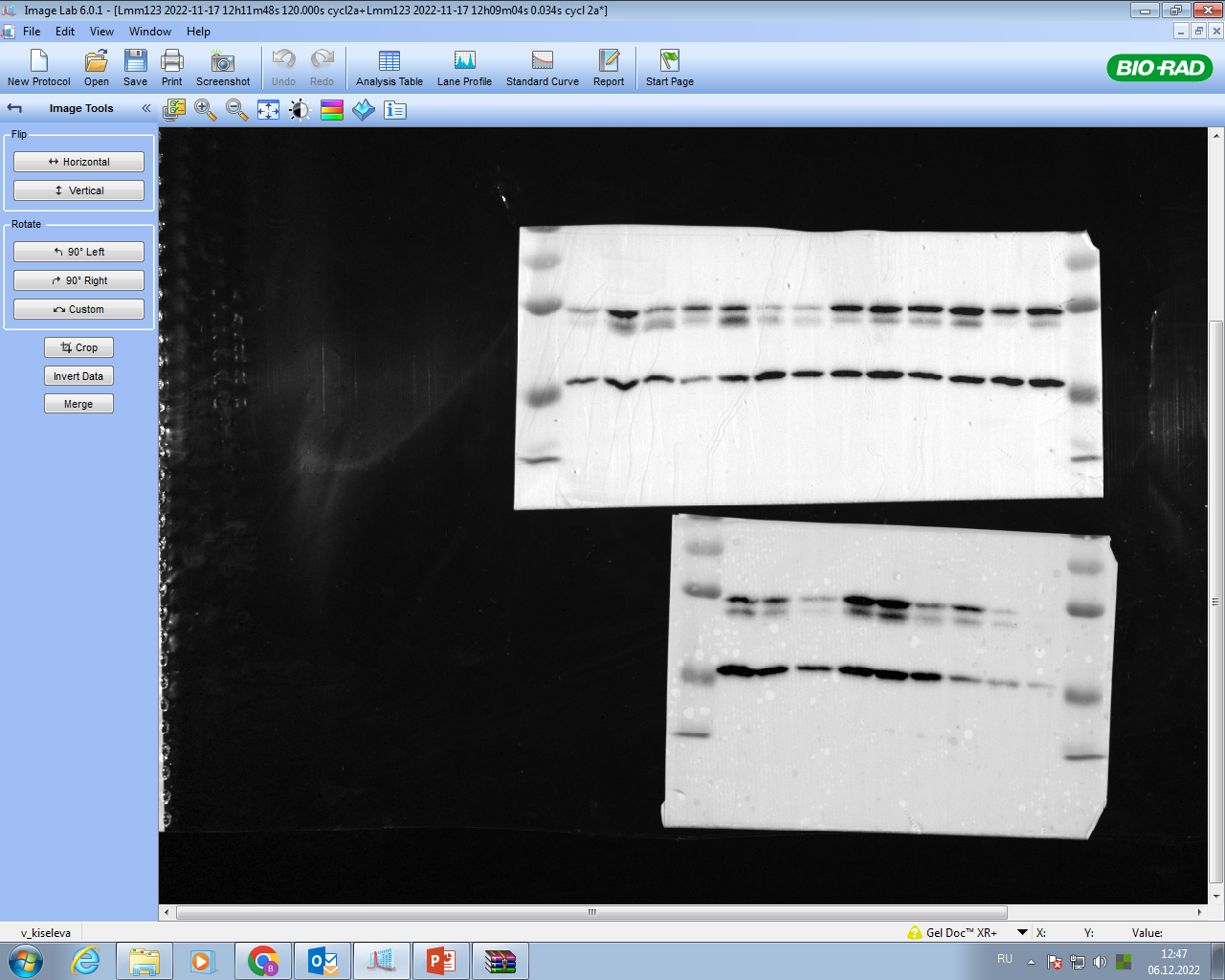


25

35

55

75

7 days

3 days

1 days

Intact spleen


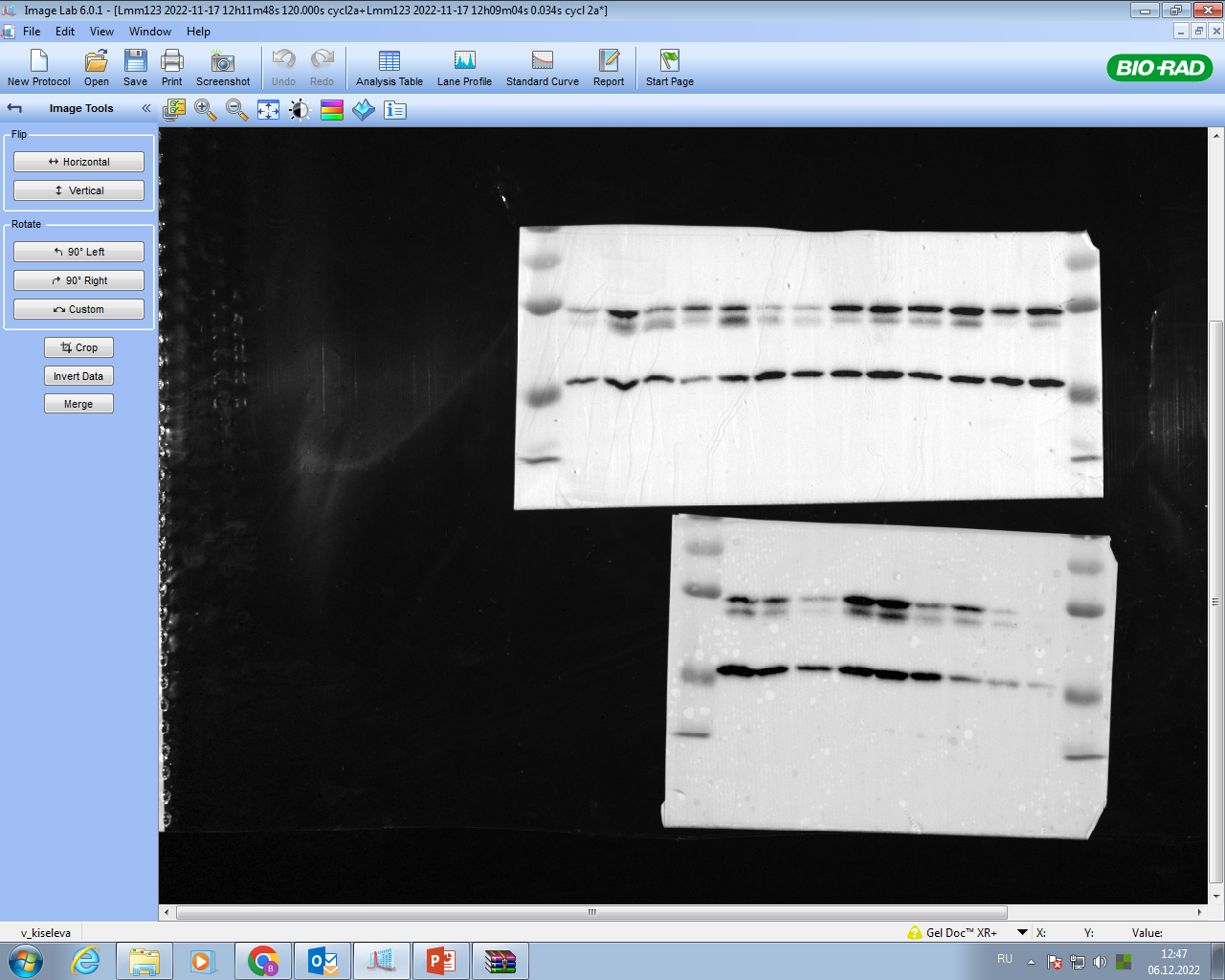


100

25

75

55

35

sham-operated

7 days

sham-operated

3 days

sham-operated

1 days

35


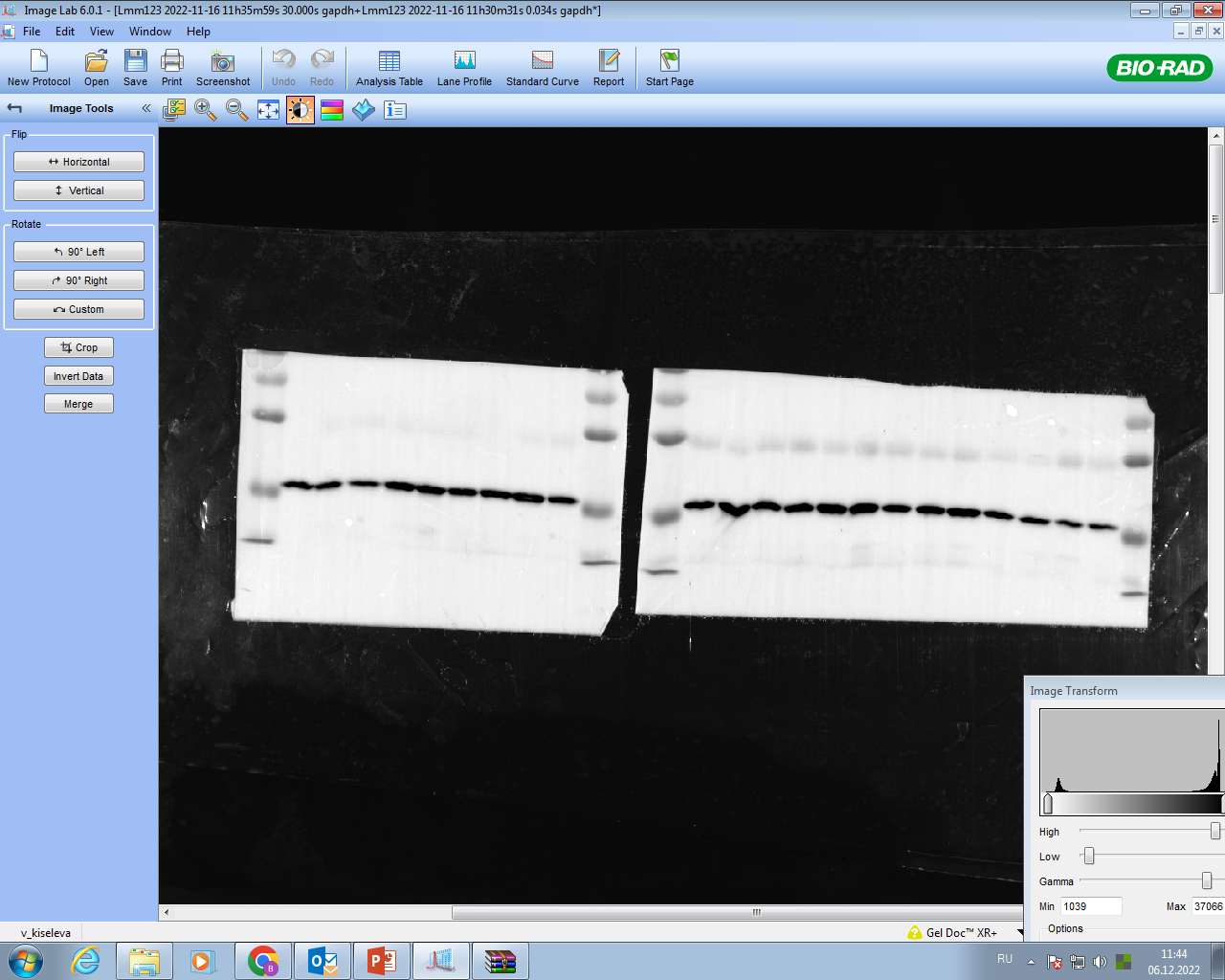


75

25

55

7 days

3 days

1 days

Intact spleen

GAPDH


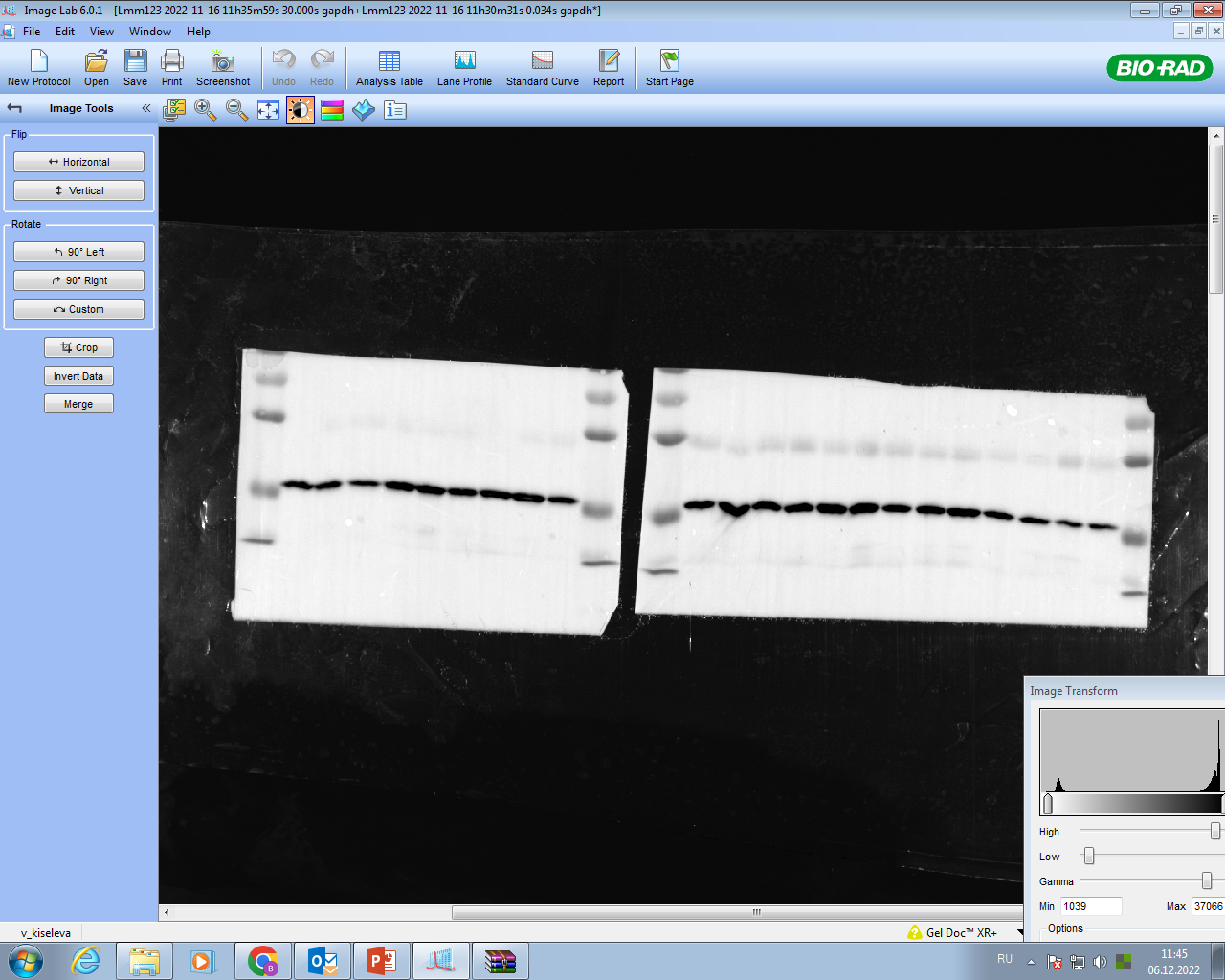


25

35

55

75

100

sham-operated

7 days

sham-operated

3 days

sham-operated

1 days


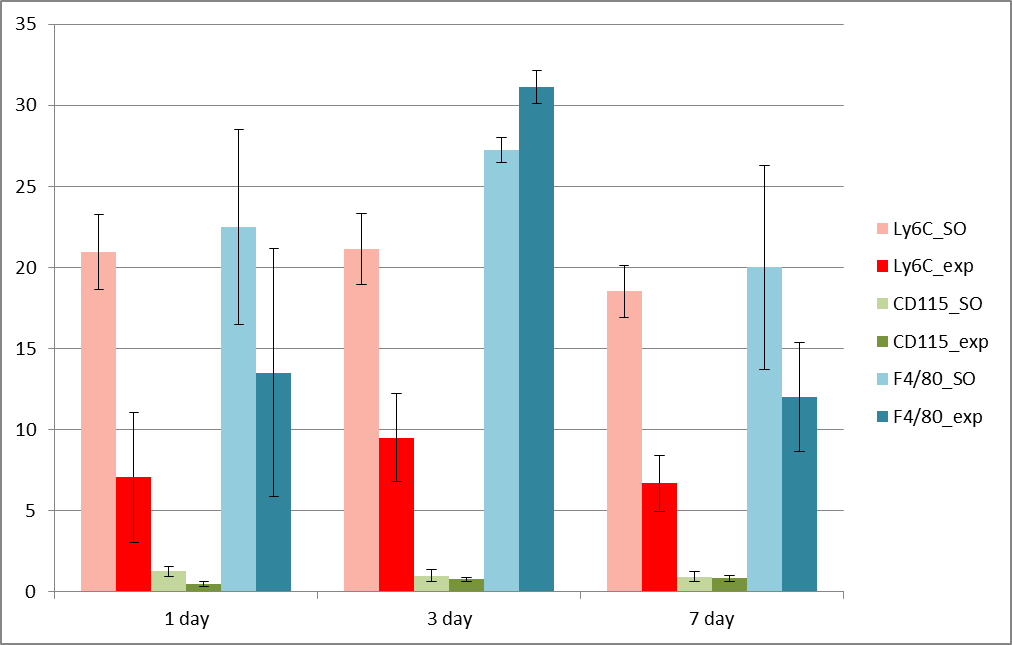


**Supplementary Figure 4.** Dynamics of spleen macrophage subpopulations during 70% liver resection in the mouse.


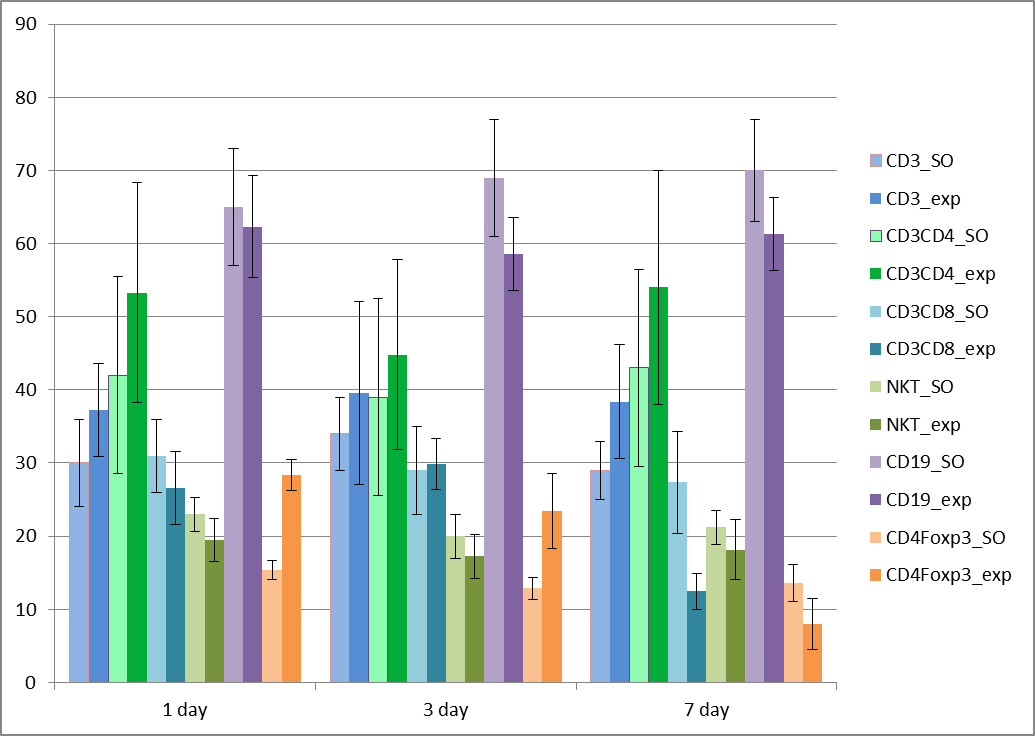


**Supplementary Figure 5.** Dynamics of spleen lymphocyte subpopulations during 70% liver resection in the mouse.
